# Supplementary figures and images for: A new method of recording from the giant fiber of Drosophila melanogaster shows that the strength of its auditory inputs remains constant with age
Source: PLoS One. 2020 Jan 7;15(1):e0224057. doi: 10.1371/journal.pone.0224057 (PMC6946141; doi:10.1371/journal.pone.0224057)

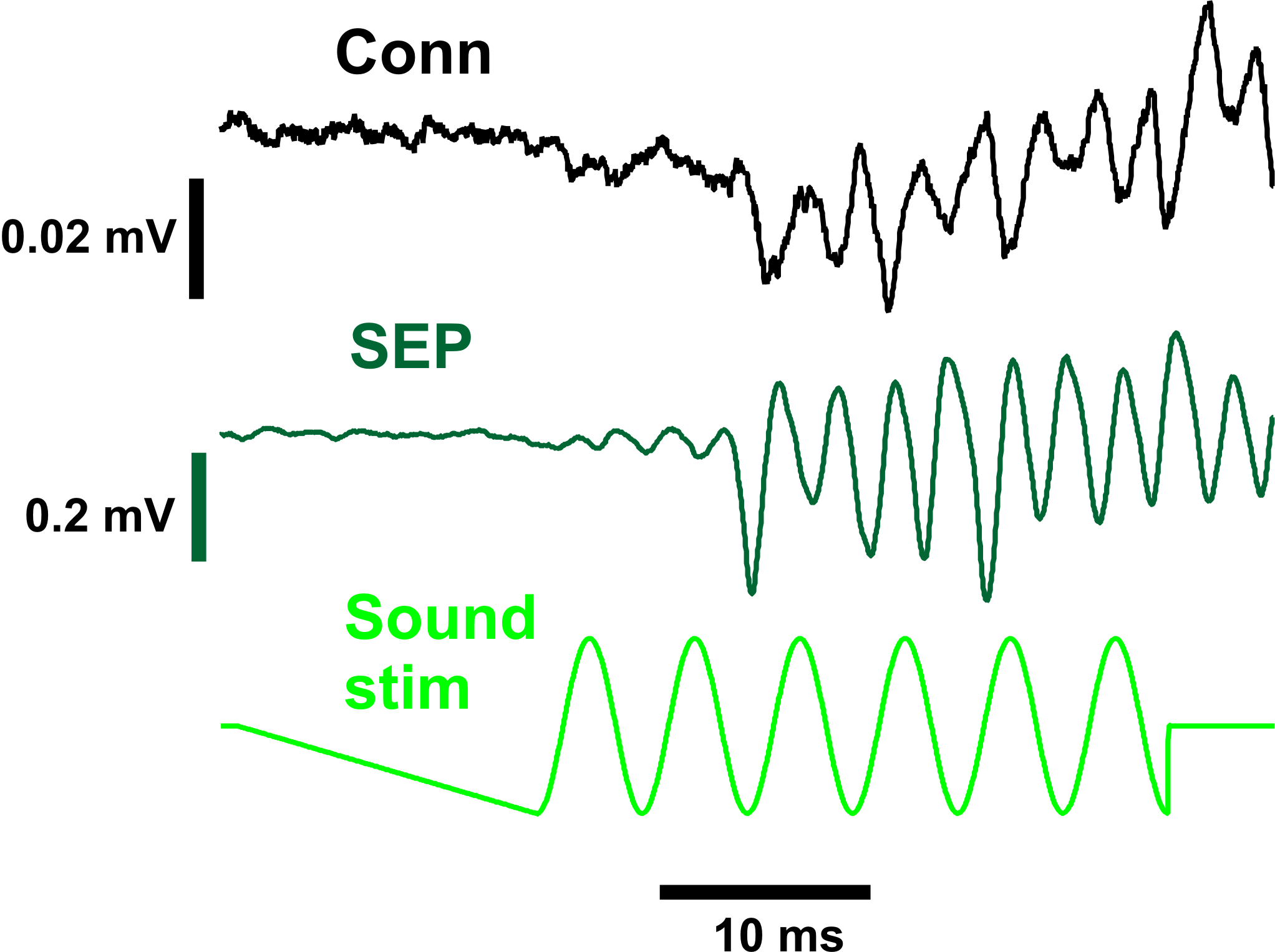

Supplement: S1 Fig — NB the sound stimulus input itself is shown, not the resultant measured sound level. (TIF) [file pone.0224057.s003.tif]
