# Supplementary material for: A new method of recording from the giant fiber of Drosophila melanogaster shows that the strength of its auditory inputs remains constant with age
Source: PLoS One. 2020 Jan 7;15(1):e0224057. doi: 10.1371/journal.pone.0224057 (PMC6946141; doi:10.1371/journal.pone.0224057)
Supplement: Attachment — Submitted filename: Response to Reviewers.docx [file pone.0224057.s005.docx]

PONE-D-19-27717 Response to Reviewers.

Line 28: Please, revise for clarity the sentence describing the 40% decrease and the following few sentences.

These sentences have been rewritten

Line 44: "motor neuron" or "motoneuron". Also line 284.

Corrected to “motor neuron”

Line 49: I think you can delete the "somewhat uncoordinated"

Deleted

Line 63: The shakeb should be designated as innexins and with a reference.

This has been done as requested

Line 70. Eliminate quotations around love song and please describe the courtship song in a sentence or two for non-Drosophila readers

As requested

Line 84: Consider using "the strength of the connection between auditory input and GF"

As suggested

Line 96: "Changes" in food probably does not mean different types of food? Please clarify in the text.

Clarified to: “changed to fresh food vials weekly”

Please, explain at line 163 the relationship between sound intensity and particle velocity. Are they linear? Some incite for readers who is not familiar with particle velocity might be beneficial.

I had incorrectly used the term “sound intensity” rather loosely here – it has a more precise meaning in acoustics, to do with power per unit area. I have changed this to “sound level” throughout. As I understand it, in the near-field conditions used here, sound particle velocity is apparently not directly proportional to sound pressure, as it is at greater distances from the source. I have made this clear in the Methods.

Line 164: Sound duration and frequency should be given. See also line 197: What does i.e. mean here? Were different frequencies used?

After further research, it appears that use of the term frequency has little meaning when applied to a sound transient or impulse, so I removed any references to the stimulus frequency, only referring to its initial peak duration and amplitude.

Line 166 and following: Maybe this could be improved a bit by explaining the general idea at the beginning of the paragraph: that an electrical stimulus activates optical pathways that are kept subthreshold, but enable the auditory stimulus to become suprathreshold and elicit GF activity. This would also help for later (line 384).

As requested.

Line 208. Remove about. The latency should be directly measured and not roughly estimated.

Done

Line 209 - 210. The phrase in the parentheses feels like a sentence fragment. Please revise it.

Done

Line 267. Remove "in passing". It's either been reported or not.

Done

Line 289. Remove the whole sentence stating that a direct measurement of GF activity would be more advantageous. It's unnecessary and such direct measurements have their own limitations (equipment expenses, more difficult, invasive, etc).

Good point. Done

Line 350. 1ms delay to what?

This was expressed badly before. I was referring to the delay from the TTL pulse at the trace onset. This has been made clearer in the text.

Line 404 / 405: Maybe this sentence would be better if split into two.

Duly split.

Split sentence in line 406 into smaller statements.

Done

Line 427: It's not clear why this is a combined results / discussion section. The discussion part clearly starts in line 427, so why not call it discussion here? (just a suggestion)

You are absolutely right – I have done as suggested.

Line 441. This half sentence needs to be moved to line 433.

Done as suggested

Figure 4. Remove the figure title. Perhaps swap it for the y-axis and use no figure title.

Good suggestion

Figure 5. There are a lot of non GFN spikes in these recordings that don't seem reliably time locked to stimulation. Perhaps mentioning that these are from other non-identified neurons should be mentioned in the figure legend.

Mentioned.

Review Comments to the Author

Reviewer #1:

1) Line 70. Eliminate quotations around love song and please describe the courtship song in a sentence or two for non-Drosophila readers

Done

2) Line 74. I don't think it's correct to refer to the adult as hatching. I believe this is eclosion.

Absolutely correct! Fixed.

3) I understand the complexity in measuring particle velocity and thus why the authors reported dB in arbitrary units. Perhaps the authors though could make a statement at line 163 about the relationship between sound intensity and particle velocity. Are they linear? Does a double in sound intensity result in at least a near doubling in particle velocity. Some incite for readers who don't think about particle velocity might be beneficial.

I had used the term “sound intensity” rather loosely here – it has a more precise meaning in acoustics, to do with power per unit area. I have changed this to “sound level” throughout. Unfortunately, in the near-field conditions used here, sound particle velocity is apparently not directly proportional to sound pressure, as it is at greater distances from the source. I have made this clear in the Methods.

4) Line 208. Remove about. The latency should be directly measured and not roughly estimated.

As above, done

5) Line 209 - 210. The phrase in the parentheses feels like a sentence fragment. Please revise it.

As above, done

6) Figure 4. Remove the figure title. Perhaps swap it for the y-axis and use no figure title.

Done

7) Line 267. Remove "in passing". It's either been reported or not.

Done

8) Line 289. Remove the whole sentence stating that a direct measurement of GF activity would be more advantageous. It's unnecessary and such direct measurements have their own limitations (equipment expenses, more difficult, invasive, etc).

Done

9) Figure 5. There are a lot of non GFN spikes in these recordings that don't seem reliably time locked to stimulation. Perhaps mentioning that these are from other non-identified neurons should be mentioned in the figure legend.

Done

10) For the diptheria toxin experiments, was there any independent validation that the toxin killed these neurons? Co-expression of GFP with the toxin is a reasonable what to do this.

This would have been the most elegant way to show that the GF was completely missing and I was originally planning to do this. However the loss of many stocks after hurricane Maria prevented me from doing these particular experiments. I did however look at the neck connective from the diphtheria toxin expressing animals and was unable to see the GF axons with Nomarski optics, when they are normally very prominent. I have now mentioned this in the Results. It must be pointed out that it is not necessary that the GF be completely eliminated, just poisoned, in order for it to stop spiking. I have also altered the word “ablated” to “poisoned”.

11) The sentence in line 406 is a very long run on sentence and was difficult to understand. Simply chop it up into smaller more comprehend-able statements.

Done

12) Line 439, eliminate "never". Just state, "because JON PSP's do not go above threshold in this configuration ...

Done

13). Please revise the writing of point (5) starting on line 439 of the manuscript. It is a really long sentence and it causes confusion. I am not sure that somatic recordings are very useful for assuming spike thresholds as the spike initiation site might be very far from the soma. I simply did not understand point (5).

This part has been rewritten, and the threshold estimate moved to point 1. The point about the actual value of spike threshold is well taken, however, all these estimates are made from the point of view of the soma, since our only measurements of EPSPs come from patch recordings.

Reviewer #2:

Line 28: The sentence describing the 40% decrease is difficult to understand. Similarly, the following few sentences could be better. They make sense once one has read the whole paper, but as a first time reader they are difficult. The end of the introduction gives a nicer summary of the results, for example.

This last part of the abstract has been rewritten to hopefully be clearer.

Line 44: "motor neuron" or "motoneuron". Also line 284.

Corrected

Line 49: I think you can deleted the "somewhat uncoordinated"

Deleted

Line 63: The shakeb should be designated as innexins and with a reference.

This has been done

Line 84: I suggest to use "the strength of the connection between auditory input and GF"

As suggested

Line 96: "Changes" in food probably does not mean different types of food? Please clarify in the text.

Clarified to mean fresh food vials weekly

Line 164: Sound duration and frequency should be given. See also line 197: What does i.e. mean here? Were different frequencies used?

After further research, it appears that use of the term frequency has little meaning when applied to a sound transient or impulse, so I removed any references to the stimulus frequency, only referring to its initial peak duration and amplitude.

Line 166 and following: Maybe this could be improved a bit by explaining the general idea at the beginning of the paragraph: that an electrical stimulus activates optical pathways that are kept subthreshold, but enable the auditory stimulus to become suprathreshold and elicit GF activity. This would also help for later (line 384).

Edited as requested

Line 350. 1ms delay to what?

This was expressed badly before. I was referring to the delay from the TTL pulse at the trace onset. This has been made clearer in the text.

Line 404 / 405: Maybe this sentence would be better if split into two.

Done

Line 427: It's not clear to me why this is a combined results / discussion section. The discussion part clearly starts in line 427, so why not call it discussion here? (just a suggestion)

They have been split into two sections, as suggested.

Line 441. This half sentence needs to be moved to line 433.

Edited

Figure 6: the averaged inset needs a scale bar and state the number of averaged responses.

Added to the figure and legend.

Figure 6B: How do we know that the large spike is not the 'usual' GF spike, while the smaller spike before is just a subthreshold reflection of synaptic input? Maybe this could be stated clearly in the text.

The small subthreshold response to eye stimulation could indeed also be from the visual circuit’s synaptic inputs to the GF, although I suspect that these are likely to be too small. I have tried to clarify this in the text.
